# Sex-Specific Automatic Responses to Infant Cries: TMS Reveals Greater Excitability in Females than Males in Motor Evoked Potentials

**DOI:** 10.3389/fpsyg.2015.01909

**Published:** 2016-01-07

**Authors:** Irene Messina, Luigi Cattaneo, Paola Venuti, Nicola de Pisapia, Mauro Serra, Gianluca Esposito, Paola Rigo, Alessandra Farneti, Marc H. Bornstein

**Affiliations:** ^1^Department of Philosophy, Sociology, Education and Applied Psychology, University of PaduaPadua, Italy; ^2^Center for Mind/Brain Sciences, University of TrentoTrento, Italy; ^3^Department of Psychology and Cognitive Sciences, University of TrentoTrento, Italy; ^4^Division of Psychology, School of Humanities and Social Sciences, Nanyang Technological UniversitySingapore, Singapore; ^5^Department of Education, Free University of BolzanoBolzano, Italy; ^6^Child and Family Research, Eunice Kennedy Shriver National Institute of Child Health and Human DevelopmentBethesda, MD, USA

**Keywords:** parenting, baby cries, MEPS, TMS, sex differences

## Abstract

Neuroimaging reveals that infant cries activate parts of the premotor cortical system. To validate this effect in a more direct way, we used event-related transcranial magnetic stimulation (TMS). Here, we investigated the presence and the time course of modulation of motor cortex excitability in young adults who listened to infant cries. Specifically, we recorded motor evoked potentials (MEPs) from the *biceps brachii* (BB) and *interosseus dorsalis primus* (ID1) muscles as produced by TMS delivered from 0 to 250 ms after sound onset in six steps of 50 ms in 10 females and 10 males. We observed an excitatory modulation of MEPs at 100 ms from the onset of infant cry specific to females and to the ID1 muscle. We regard this modulation as a response to natural cry sounds because it was attenuated to stimuli increasingly different from natural cry and absent in a separate group of females who listened to non-cry stimuli physically matched to natural infant cries. Furthermore, the 100-ms latency of this response is not compatible with a voluntary reaction to the stimulus but suggests an automatic, bottom-up audiomotor association. The brains of adult females appear to be tuned to respond to infant cries with automatic motor excitation.

## Introduction

Evolutionary theory posits that adults' responsiveness to infant-related stimuli plays a crucial role in offspring survival, and so enhances reproductive success (Darwin, 1872). In humans, responsiveness to infants ranks among the most biologically relevant and adaptive behaviors and constitutes a basis of parent-infant interaction; infant cries and caregiver responses to them are a foundation for the wholesome psychological development of the parent-child relationship and secure attachment in the individual (Bowlby, 1969; Ainsworth et al., 1978; Parsons et al., 2010). As infants command little in the way of agency, however, their cry is one of the earliest forms of communication (Zeifman, 2001; Soltis, 2004; Cecchini et al., 2007; Newman, 2007), and hearing an infant cry elicits caregiver behaviors aimed at increasing proximity with and care of the infant. Notable among such responses are motor behaviors, such as picking the infant up, applying tactile and vestibular stimulation, and talking to the infant (Gustafson and Harris, 1990; Bornstein et al., 1992). Healthy human mothers are likely to pick-up and hold and to speak to their infants in response to their infant's cry, and this specific complex of motor responsiveness is known to calm an infant (Esposito et al., 2013a).

The high biological relevance of infant-related stimuli captures adult attention and automatically triggers physiological responses that prepare adults for action (Brosch et al., 2007). For example, infant cries modulate listeners' hormonal levels (Fleming et al., 2002; Swain and Ho, 2012) and autonomic activity, as measured by heart rate, blood pressure, and skin conductance (Frodi et al., 1981; Boukydis and Burgess, 1982). At the behavioral level, preparation for action in response to infant cries has been observed in increased hand grip force (Bakermans-Kranenburg et al., 2012) and the speed and accuracy in intentional movements (Parsons et al., 2012). This motor activation may reflect an adaptive “high-alert” state that prepares adults to react rapidly to infants' distress. In accord with “embodied simulation” theory (Gallese et al., 2007), the internal simulation of observed infant behaviors, mediated by mirror neurons, enables or promotes empathic understanding in adults. Thus, internal simulation activated by baby cries may mediate parental behavior and thereby help regulate infant distress, even if evidence suggests that motor activation in response to infant cry can sometimes have negative implications in irritation and harsh parenting (Frodi, 1985; Crouch et al., 2008).

Coordinate with the autonomic and behavioral literature, functional magnetic resonance imaging (fMRI) has revealed patterns of cerebral activity following exposure to infant distress vocalizations that are associated with approach behaviors and motivation to caregiver (Barrett and Fleming, 2011; Swain, 2011; Swain et al., 2011; Pechtel et al., 2013). Germane to the present study, activity in the premotor cortex, prominently in the supplementary motor area and the dorsal premotor cortex, increases in response to infant cries (Montoya et al., 2012; Musser et al., 2012; Venuti et al., 2012; De Pisapia et al., 2013). Activation in these areas, which are part of the mirror system (Rizzolatti et al., 2001; Rizzolatti and Craighero, 2004; Cattaneo and Rizzolatti, 2009), has been interpreted as obligatory preparation for motor responses to the expression of infant need. With regard to the involvement of mirror neurons in audition, mirror neuron theory has been invoked to explain the close interaction between perception and action, such as motor theory of speech (Liberman and Mattingly, 1985). Such theories reinforce the expected close interaction between cry perception and parental action.

Given the relatively protracted time-course of hemodynamic responses, however, on the sole basis of fMRI data it is not possible to determine whether preparation for motor responses is automatic. Furthermore, it is not possible to tell if fMRI-indicated excitability translates into motor preparation. To test hypotheses of rapid motor involvement and responsiveness to infant cry more directly, we investigated motor evoked potentials (MEPs) to infant cries via transcranial magnetic stimulation (TMS).

One physiological signature of automatic bottom-up responses is their rapid onset to stimulation. It has been shown in multiple domains of perception/action that automatic sensorimotor associations can be observed in the very earliest phases of stimulus processing, that is between 100 and 250 ms following stimulus onset (see below). Later appearing responses typically reflect the expression of top-down executive control on stimulus-response associations, as obtains for most domains of sensorimotor behavior, such as saccades performed during a visual search (Van Zoest and Donk, 2006), hand movements toward graspable objects (Goslin et al., 2012), spatially oriented movement (Michelet et al., 2010), action mirroring (Barchiesi and Cattaneo, 2013; Ubaldi et al., 2015), and phonological-articulatory matching to speech sounds (Roy et al., 2008).

The hypothesis that affiliative stimulation automatically evokes preparation for motor responses therefore calls for an empirical demonstration based on a method that has a high temporal resolution. Such an approach could also disentangle bottom-up automatic responses from top-down cognitively mediated ones. For example, evidence for automatic reactions to affiliative stimuli has been provided using event related brain potentials (ERPs; Maupin et al., 2015). The N100 differs in female participants listening to infant cries compared to control stimuli (Purhonen et al., 2001, 2008), and a difference emerges in midbrain local field potential only 49 ms after hearing infant vocalizations compared to control sounds (Parsons et al., 2014b). Cumulatively, this research points to specific, automatic activity in response to infant cries that may reflect the initiation of a state of alertness necessary to activate caregiving.

In the present experiments, we used event-related TMS, which can help to disentangle automatic responses from cognitively mediated ones because its temporal resolution in detecting changes of excitability within the motor cortex falls in the range of ms. At the same time, the spatial resolution of TMS allows specific localization of activation in brain areas involved in motor responses. ERP approaches are not so spatially accurate. Furthermore, TMS (*contra* ERP) is particularly well suited to testing motor responses, of specific interest here. Increased excitability of motor cortex in response to generic emotional stimuli has been reported in previous TMS studies, most using emotional pictures (Hajcak et al., 2007; Schutter et al., 2008; Coombes et al., 2009; van Loon et al., 2010; Borgomaneri et al., 2014). Very few studies have extended this paradigm to show increased excitability of motor cortex to auditory stimuli, such as emotional sounds (Komeilipoor et al., 2013), emotional music (Baumgartner et al., 2007), and emotional spoken scenarios (Baumert et al., 2011). To the best of our knowledge, our study is the first to investigate automatic preparation for motor responses to infant-related auditory stimuli.

We applied single-pulse TMS (spTMS) to participants' motor cortex, time-locked to the auditory presentation of infant cries, and we simultaneously recorded MEPs. MEP amplitude resulting from spTMS allows the quantification covert motor preparation. The extant literature indicates that frequent and universal responses to baby cries are picking up and holding, both motor patterns (Gustafson and Harris, 1990, p. 144). Therefore, we investigated proximal and a distal muscles involved in such behavioral responses: the interosseus dorsalis primus (ID1) and the biceps brachii (BB).

We developed several *a priori* hypotheses, which we tested in two companion experiments. Due to the biological relevance of such motor behavior, we first hypothesized that motor responses will be activated automatically by baby cries. On account of its fine temporal resolution, TMS allowed us to test this hypothesis by measuring and detecting changes in excitability within the motor cortex over very brief durations. With this aim, we recorded MEPs produced by TMS delivered from 0 (a baseline condition for MEPs at a moment when the brain could not yet have access to the auditory information) to 250 ms from sound onset. We did so in six steps of 50 ms to trace the time course of the MEP with enhanced accuracy.

Second, previous studies have provided mixed evidence on gender differences in responsiveness to infant cry. Boukydis and Burgess (1982) reported gender differences in perceptions of infant cry; Byrd-Craven et al. (2015) reported that infant crying is a more potent stressor and increases cortisol in women more than in men; Out et al. (2010) and Tkaczyszyn et al. (2013) reported that women listening to baby cry show differentpatterns of cardiac sensitivity compared to men; and in fMRI investigations, compared to males, females hearing baby cries show stronger activation in amygdala and anterior cingulate cortex (Sander et al., 2007), and decreased activity the medial prefrontal cortex, suggesting that baby cries interrupt their on-going mind-wandering (Seifritz et al., 2003; De Pisapia et al., 2013). Others have reported no gender differences in ratings of motivation and arousal levels in response to baby cries or valence of baby cries (Leger et al., 1996; Parsons et al., 2014a), suggesting that responsiveness to infant cries may be related to caregiving responsibilities that parents report they assume (Donate-Bartfield and Passman, 1985). To clarify this issue, we tested a hypothesis concerning the effect of gender on automatic responses to baby cries by comparing males and females.

Third, fundamental frequency (f0) is one of the most important acoustic characteristics of baby cry (Lester and La Gasse, 2008; Esposito and Venuti, 2010a,b), and it has been shown to govern caregiver perceptions and responses (LaGasse et al., 2005). Specifically, episodes of crying with higher f0 are perceived as more negative (Gustafson and Green, 1989; Zeifman, 2003), even by members of different ethnic groups (e.g., Japanese and European listeners; see Esposito et al., 2012, 2013b, 2015). Considering the importance of f0 as a prominent acoustic characteristic of baby cry, we hypothesized that automatic preparation for motor responses would be specific for f0. To evaluate this hypothesis, we tested preparation for motor responses while listening natural baby cries in comparison to acoustically modified baby cries (systematically varied in f0).

## General methods

### Participants

All participants were young healthy adults who gave written informed consent and were screened for contraindications to TMS (Rossi et al., 2009). The two experiments reported here were approved by the Ethical Committee of the University of Trento and conducted in compliance with the revised Helsinki declaration (World Medical Association General Assembly, 2004).

### Procedures

With exception of the stimuli, the experimental procedures were identical in the two experiments.

#### Main experiment: Participants and experimental design

Twenty healthy right-handed, participants (10 Females, 10 Males, *M* age females = 28.3 years, *M* age males = 32.1 years) took part in the main experiment. They were tested during a single 40-min session in which they listened passively to cry sounds that were periodically delivered through earphones. Single-pulse TMS was systematically delivered at different inter-stimulus intervals (ISIs) from the onset of each acoustic stimulus (0, 50, 100, 150, 200, and 250 ms). MEPs to spTMS were recorded and were the source of the main dependent variable after data processing. Each participant underwent 270 trials (6 ISIs × 3 cry types; see below). Each cell of the experimental design therefore contained 15 repeated trials. Power analysis showed that we could detect medium to large effects (effect size range = 0.5–0.8) employing F-test family statistics on independent groups with a *p*-value set at 0.05.

#### Main experiment: Auditory presentations

Acoustic stimuli were presented using E-Prime 2.0 software. They were recorded from natural baby cries generated from a digital audio file of the cries of a 6-month-old boy before a scheduled feeding. The infant was born term and showed no signs of any clinical conditions at birth or at age 3 years. Five 250-ms cries were cropped from the initial part of 5 different cry episode of the child and were selected for their typical rhythmic quality (natural cry segments). All cry stimuli were normalized for intensity, and the volume was kept constant for all the presentations for all the participants. A long-term average spectrum (LTAS) provided spectral information for each cry. For all 250-ms cry segments, f0 of the LTAS was obtained. Mean f0, the frequency value (in Hz) of the first amplitude peak across the LTAS, was 502.14 Hz (SD = 25.6) for natural cry segments. Subsequently, natural cry segments were experimentally manipulated employing Praat software for audio editing (Boersma, 2002, ver. 5.0.06). Two groups of five cry segments with f0 augmented 200 Hz (+200 Hz) and 400 Hz (+400 Hz) were produced. The three cry types (natural cry, +200 Hz, and +400 Hz) were then presented randomly, at irregular ISIs to avoid anticipatory responses. Each stimulus was presented 24 times.

#### Main experiment: TMS

SpTMS was delivered with a biphasic Magstim Rapid (Magstim, Dyfed, UK) stimulator connected to a standard figure-of-eight coil with an outer winding diameter of 70 mm. The coil was positioned with the handle pointing backward at 45° from the midline over the optimum scalp location where MEPs with the maximal amplitude could be obtained from the BB and 1DI muscles at minimum stimulus intensity. Motor thresholds (Rossini et al., 1994) are commonly used to individually adjust the intensities of TMS. In the main experiment, the topographic location on the cortex and the basal excitability of representations of the two muscles were so different that, rather than using a single muscle as a target for motor threshold determination, we opted for a stimulation intensity at which MEPs with amplitudes between 500 and 1500 uV were evoked from both muscles.

#### Main experiment: Electromyographic recording and processing of MEPs

The EMG signal from each participant's right upper limb was collected by means of two pairs of surface Ag/AgCl electrodes positioned on the skin of the dominant arm overlying the belly and tendon of the *biceps brachii* (BB) and *interosseus dorsalis primus* (ID1) muscles and connected to two analog amplifier channels (CED 1902 unit—Cambridge Electronic Design, UK). The signal was amplified 1000x and digitized (4 KHz sampling rate) by means of a CED power 1401 analog-to-digital converter, controlled by the Signal software (Cambridge Electronic Design, UK). Recordings were digitally band-pass filtered between 20 Hz and 2 KHz with a notch filter at 50 Hz. We extracted the peak–peak amplitude of MEPs from each of the two EMG channels and used it to produce the main experimental dependent variable. We also collected minimum and maximum values of spontaneous activity in the 100 ms preceding the MEP to check for voluntary muscular activity defined as maximum–minimum activity exceeding 50 μV on either of the 2 EMG channels. Trials with voluntary activity were excluded from further analysis. Stimulation intensities determined in this way ranged between 43 and 72% of stimulator output. Voluntary activity in the pre-stimulus period over a 200-ms interval was assessed visually. Single trials with EMG activity exceeding 100μV were excluded from further analysis. On average across participants, 6% of trials were excluded.

As a *post hoc* control, we pooled recorded MEP amplitudes of all participants and found average values of 0.82 mu (± 95% CI: 0.19) for the BB muscle and 1.22 mu (± 95% CI: 0.37) for the ID1 muscle.

#### Data analysis

Motor evoked potential amplitudes were calculated as positive peak-negative peak amplitudes. Raw MEP amplitudes from all participants in the main and control (see below) experiments were analyzed separately for the two muscles. First, raw MEP amplitudes were standardized within each muscle as z-scores by subtracting the grand-average of the MEPs from each MEP for that muscle and dividing the difference by the standard deviation of the population of MEPs from the same muscle. The *z*-scores were then averaged within each cell of the design so that each participant contributed 36 data cells (18 data cells for each of the two muscles). All variables were normally distributed. The data from the main experiment were analyzed as dependent variables in two ANOVAs (one for each of the 2 muscles) with one between-subjects factor, SEX (2 levels, male or female) and 2 within-subjects factors, ISI (6 levels: the 0, 50, 100, 150, 200, and 250 ms) and CRY (3 stimulus types; natural baby cries, +200 Hz, +400 Hz). Due to the repeated-measures design, the variable subject was included as a random effect.

The control experiment was performed *post hoc* to test the specificity of findings of the main experiment. It was analyzed separately from the main experiment because the grouping variables were not homogeneous. In the main experiment, participants were grouped according to sex. In the control experiment, participants were grouped according to the type of acoustic stimuli they heard. The data from the control group of 10 females listening to scrambled cries were therefore analyzed together with the group of 10 females in the main experiment. This was done by means of two ANOVAs (one for each of the 2 muscles) with one between-subjects factor, SOUND (2 levels, original cries or scrambled cries) and 2 within-subjects factors, ISI (6 levels: the 0, 50, 100, 150, 200, and 250 ms) and CRY (3 stimuli types; natural baby cries, +200 Hz, +400 Hz). Finally, in both experiments, significant effects were explored in planned comparisons consisting of pairwise *t*-tests between the data from the 0 ms ISI (baseline) and the other 5 ISIs.

## Results

### Main experiment

Univariate distributions of the dependent variables were examined for normality, homogeneity of variance, outliers, and influential cases; normality prevailed (Tabachnick and Fidell, 2001).

**Figure 1:**
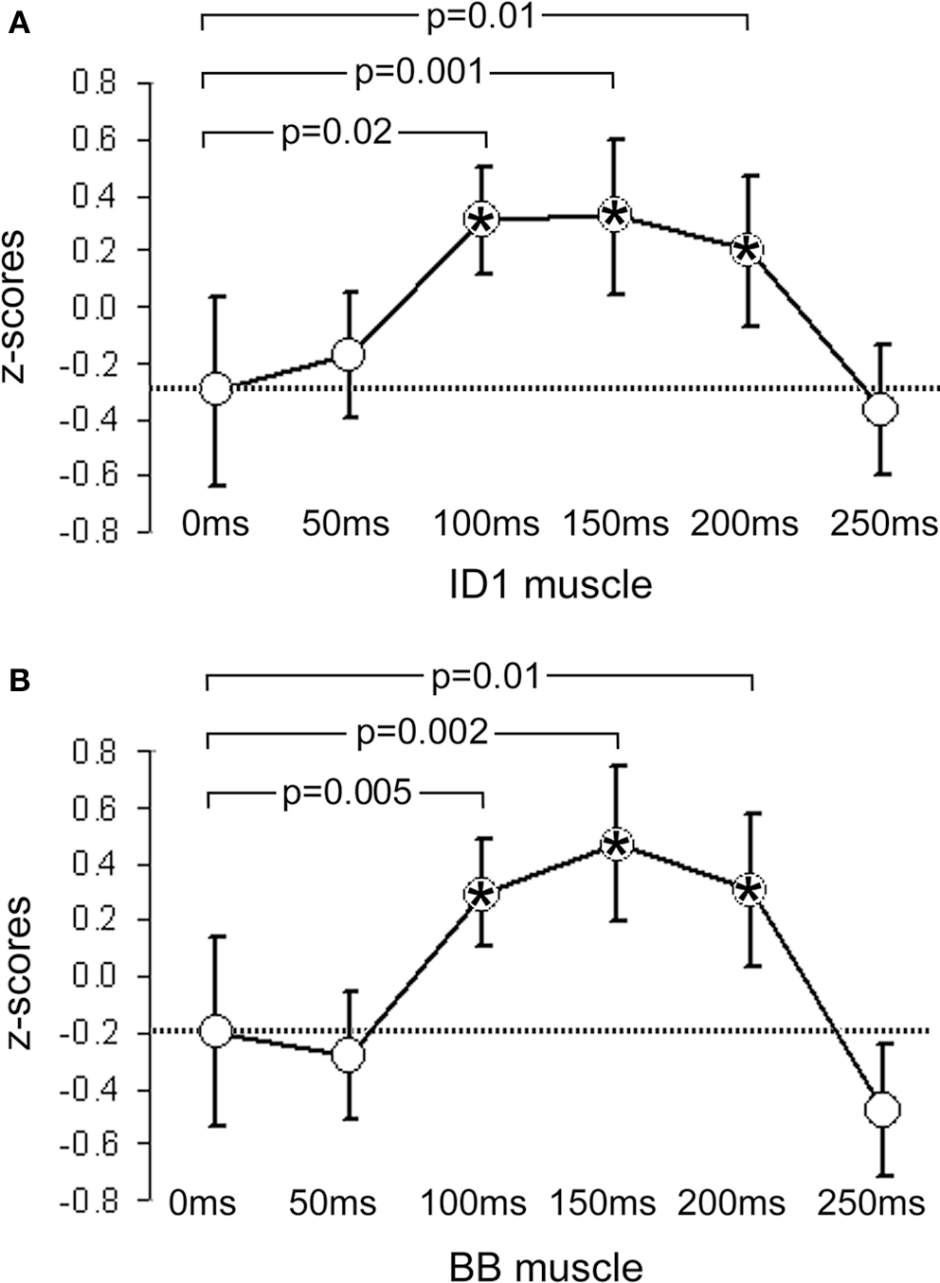
**Mean *z*-scores pooled from all participants and across all cry types**. **(A)** shows data from the ID1 muscle and **(B)** data from the BB muscle. Bars indicate 95% CIs. The dashed line represents the mean value obtained at 0 ms (baseline). *P*-values indicate significant differences between the data obtained at 0 ms and the data obtained at the other 5 ISIs. Significant time-points are indicated with asterisks.

**Figure 2:**
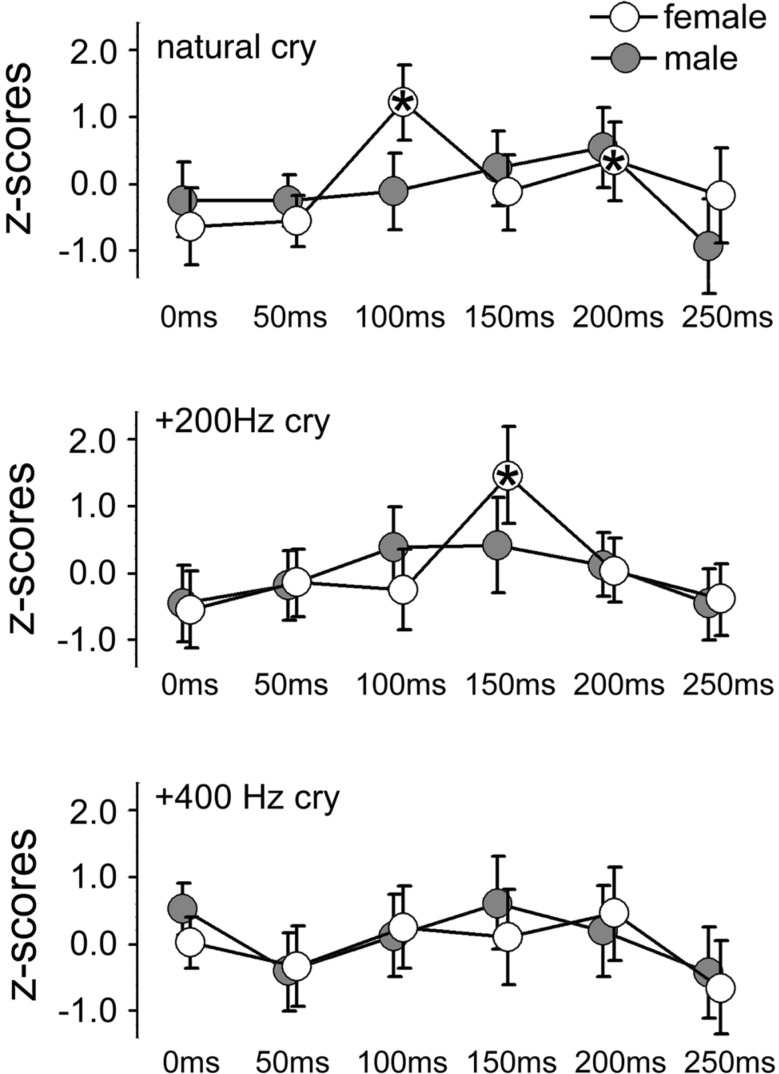
**Mean *z*-scores for each cry type and each ISI for females and males**. Bars indicate 95% CIs. Asterisks indicate ISIs at which a significant difference was found with data obtained at 0 ms (baseline).

In the ID1 muscle, specific results emerged: only females (not males) listening to baby cries (not to control sounds) produced increases in MEP amplitudes, and only when the sound-TMS interval was 100 ms. A similar finding emerged with slightly delayed MEP increase (150 ms) when sounds were slightly modified (in the +200 Hz condition). The ANOVA on the ID1 muscle data showed a main effect of ISI, *F*(5, 90) = 9.29, *p* = 0.0000004, η^2^ = 0.15, illustrated in Figure 1A, and a SEX × ISI × CRY 3-way interaction, *F*(10, 180) = 2.80, *p* = 0.003, η^2^ = 0.07, illustrated in Figure 2. To investigate this interaction, the design was split into two ISI × CRY ANOVAs, each with data from one sex. The analysis of males yielded only a main effect of ISI, *F*(5, 45) = 4.1570, *p* = 0.003, η^2^ = 0.13; type of stimulus was not significant, *F*(2, 18) = 0.32, *p* = 0.73. By contrast, the analysis performed on females showed a main effect of ISI, *F*(5, 45) = 6.02, *p* = 0.0002, η^2^ = 0.19, and a 2-way ISI × CRY interaction *F*(10, 90) = 5.23, *p* = 0.000005, η^2^ = 0.21. Three separate one-way ANOVAs for each of the 3 cry types for females showed significant effects for the natural cry, *F*(5, 45) = 6.78, *p* = 0.00009, η^2^ = 0.39, and for the +200 Hz cry, *F*(5, 45) = 9.22, *p* = 0.000004, η^2^ = 0.47, but not for the +400 Hz cry, *F*(5, 45) = 1.95, *p* = 0.11, η^2^ = 0.14. Three significant results emerged from planned comparisons: significant deviations from baseline (0 ms ISI) at 100 ms (*p* = 0.001) and 200 ms (*p* = 0.004) ISIs for the natural cry data indicated, and a significant difference from baseline at 150 ms ISI (*p* = 0.001) for the +200 Hz cry.

In the BB muscle, no responses specific to baby cries were elicited in males, *F*(2, 18) = 2.31, *p* = 0.13, or females, *F*(2, 18) = 1.23, *p* = 0.32. Only a non-specific increase in MEP amplitude with increasing sound-TMS delays was found. The ANOVA on the BB muscle data showed only a main effect of ISI, *F*(5, 90) = 5.34, *p* = 0.0002, η^2^ = 0.09, illustrated in Figure 1B, and no significant results emerged in the ISI × CRY interaction in either males, *F*(10, 90) = 0.84, *p* = 0.59, or females, *F*(10, 90) = 0.55, *p* = 0.85.

### Control experiment

To better control for the specificity of infant cries to elicit sex-specific automatic preparation for motor responses, we undertook a control experiment in which a different group of 10 healthy, right-handed females (*M* age = 27.7 years) were exposed to cry and control stimuli. The auditory control stimuli were obtained by scrambling the natural and pitch-modified baby cries used in the main experiment. Scrambling was realized following procedures proposed by Collignon et al. (2015) that maintain similar low-level physical features in original and scrambling sounds, inspite of scrambled sounds becoming completely unrecognizable (Collignon et al., 2015; Dormal et al., 2015). Scrambled versions of the sounds were performed in MATLAB (The MathWorks, Inc., Natick, MA, USA). Each sound was submitted to a fast Fourier transformation, and the resulting components were separated into frequency windows of ~700 Hz based on their center frequency. Scrambling was then performed by randomly intermixing the magnitude and phase of each Fourier component within each frequency window separately. The inverse Fourier transform was then applied to the resulting signal. The output was a sound of the same length of the original sound with similar energy within each frequency band. Following standard practices, sounds and their scrambled versions were equalized in root mean square (RMS) level.

**Figure 3:**
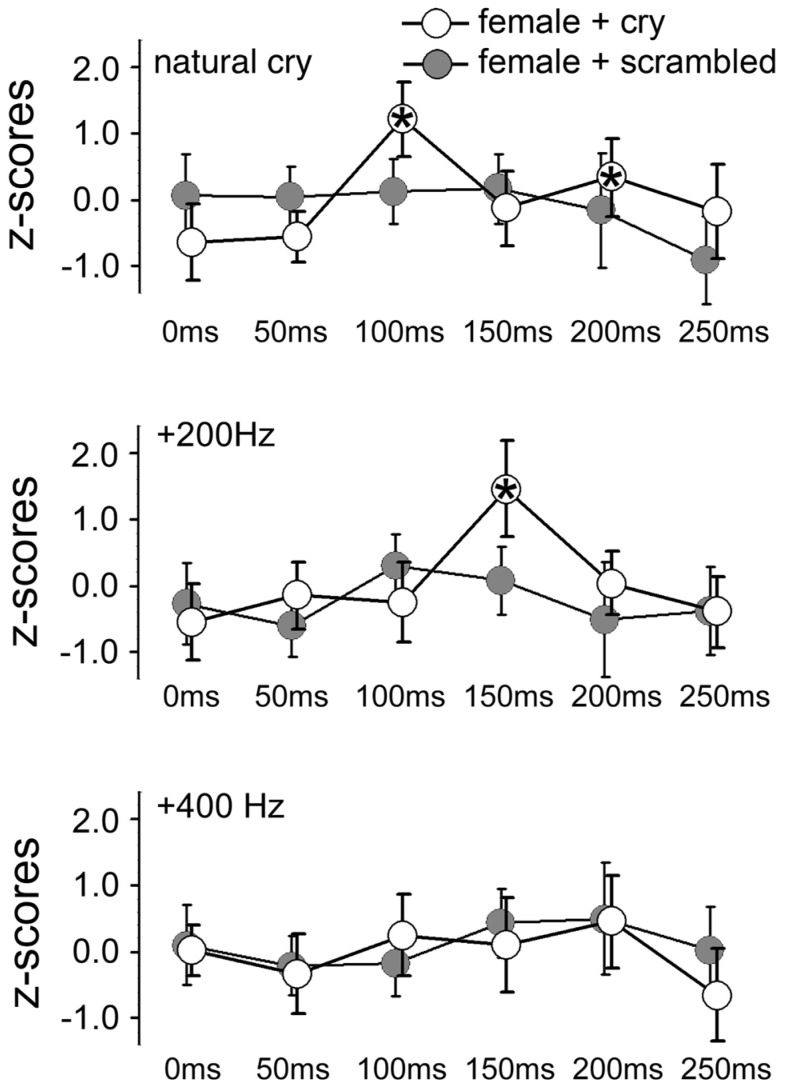
**Mean *z*-scores for each sound type and each ISI in the two groups of females (main and control experiments)**. Bars indicate 95% CIs. Asterisks indicate ISIs at which a significant difference was found with data obtained at 0 ms (baseline).

The ANOVA on data from the BB muscle showed only a main effect of ISI, *F*(5, 90) = 5.31, *p* = 0.0003, η^2^ = 0.08. The ANOVA on data from the ID1 muscle yielded a significant main effect of ISI, *F*(5, 90) = 5.76, *p* = 0.0001, η^2^ = 0.09, and a 3-way interaction of GROUP × ISI × CRY F(10, 180) = 2.67, *p* = 0.005, η^2^ = 0.08, as illustrated in Figure 3. The same strategy as in the Main Experiment was used to analyze this interaction. A 2-way ISI × CRY ANOVA of women who listened to scrambled cries showed no significant interaction, *F*(10, 90) = 0.76, *p* = 0.67. Results for the women who listened to the original (un-scrambled) cries are reported in the Results of the main experiment.

## General discussion

In the present studies, we found that the corticospinal system of adults (non-parents) is modulated by exposure to the sound of infant cries. Specifically, in support of our first and second hypotheses, we observed rapid facilitatory effects in one of two recorded muscles, present only for natural infant cry sounds, and occurring only in females. To set these conclusions in context, it is useful first to describe the general non-specific response to any auditory stimulus that is normally detectable in both muscles of participants of both genders (Figure 1). The temporal characteristics of this response are compatible with facilitation of the TMS-evoked corticospinal volley by a concomitant auditory startle, known to appear in the biceps at latencies beyond 55–60 ms (Brown et al., 1991b). However, two factors indicate that the non-specific arousal response which we recorded is not attributable exclusively to classical auditory startle. First, the stimulation applied in the present study was sufficiently frequent to habituate a startle response within the first few trials (Brown et al., 1991a). Second, the auditory startle response is usually more evident in proximal than in distal muscles (Brown et al., 1991b), which was not the case in the present results.

As shown in Figure 2, we found preparation for motor responses that were specific to females and that were present as early as 100 ms following auditory stimulation by natural infant cries. What do these features of the audiomotor response tell us? First, its latency strongly implicates an automatic audiomotor association, considering that simple auditory reaction times in the distal upper limbs center around 200 ms (Ritter et al., 1972). Given the latency of 100 ms from the onset of auditory stimulation, it is not clear where the response originated. It could be mediated by a wholly subcortical circuit, as in the auditory startle response discussed above, or in orientation responses to auditory stimuli mediated by brainstem pathways passing through the tectal and pretectal regions. Another possible subcortical node that mediates such fast audiomotor associations is the amygdala, which is known to be involved in parental caregiving (Barrett et al., 2012) and is a subcortical center that mediates fast visuomotor associations in other affective domains (Sah et al., 2003). However, latencies on the order of 100 ms are compatible with an early transcortical response (Martin et al., 2007). These explanations are not mutually incompatible as subcortical and cortical contributions to the TMS response could coexist.

A second feature of our results is their specificity to natural infant cries. The earliest facilitatory peak we observed appeared at 100 ms in response to such cries, although a similar facilitatory peak was found in association with +200 Hz cry but at slightly longer (150 ms) latency (compare the top and middle panels of Figure 2). No peak at all was observed in response to the +400 Hz transformed cries. Therefore, in agreement with our third hypothesis, audiomotor effects were elicited specifically by baby cries around their natural frequency. With regard to the observation of a peak with longer latency in association with +200 Hz cry, a well-established association between pitch in infant cries and perceived distress (Donovan et al., 1998; Schuetze and Zeskind, 2001; Young et al., 2012) suggests that motor preparation is likely associated with an aversive response. Future studies might focus on this hypothesis comparing adult responses to baby cries with less dramatic manipulations of the natural frequency (in our study the +400 Hz transformed cries were clearly not perceived as a baby cries).

This finding could reflect non-specific startle effects of sounds in that particular frequency range. The control experiment on a separate group of women listening to the same sounds but scrambled by means of a procedure that keeps constant the frequency and intensity envelope of the sounds also showed an automatic response specific to natural baby cries.

A third feature of the audiomotor response described here is its sex-specificity and restriction to women. This specificity is unlikely to depend on general gender differences in response to emotional vocalization, because the latter have been reported to be small in extant literature (Belin et al., 2008; Parsons et al., 2014b). Thus, the present results point to a sex dimorphism specifically in response to infant cries. This interpretation is consistent with earlier studies that reported sex-differences in brain responses to baby cries (Seifritz et al., 2003; Sander et al., 2007; De Pisapia et al., 2013). The absence of an early audiomotor response in male listeners in this experimental paradigm should not be taken as evidence of the absence of an infant-sensitive neural system in males, however. Other factors, such as a higher threshold or increased habituation to repeated stimuli in males versus females, could account for the present differential results (Andreano et al., 2014). These alternatives do not, however, negate the sex-specificity we found.

A fourth feature of the results that merits discussion is that the sex difference in responsiveness to baby cries we found should be considered in light of the non-parent status of the participants. Indeed, evidence for sex differences in preparation for motor responses to baby cries has been found in non-parents, whereas no sex differences have been found between male and female parents in some physiological measures, such as skin conductance and heart rate in response to baby cries (Frodi et al., 1978a,b). As our participants were not parents, the audiomotor responses we recorded were probably not the product of extensive associative experience with infant cries, as could be the case with parents (see Limitations below). Indeed, important behavioral, physiological, and endocrine changes occur in women and in men when they become parents (Storey et al., 2006; Delahunty et al., 2007; Bornstein, 2015), and sex differences in brain activation in response to baby cries have been found to change fundamentally with parental experience (Seifritz et al., 2003). Such changes are associated with social context variables, such as contact with children (Storey et al., 2011). Moreover, finding automatic responsiveness to baby cries in nullipara women lends further support to the idea of an “alloparental care” predisposition in females (Hrdy, 2007), but not in males, similar to several mammalian species which feature cooperation in infant care (Briga et al., 2012).

Last, the muscle specificity of the response we observed is more challenging to interpret because it requires the formulation of a hypothesis in which specific behavior is elicited in females that involves distal hand muscles more strongly than proximal ones. The data collected in this experiment also do not speak to a whole-body preparation for motor responses, because we recorded from only two muscles. One explanation might be that the muscular pattern we observed indicates a propensity to reach for the infant and so is expressed as an extensor (rather than a flexor) response. It could be that facilitatory responses recorded in the ID1 muscle are a biological marker of a stimulus-response association or an index of a specific motor pattern.

On the basis of human and animal studies investigating brain responses to infant signals, a neurological model of the parental brain has been advanced (Swain et al., 2014). In that model, cues specific to infants (e.g., cries, laughs, images, touch, or odors) activate subcortical structures that promote the salience of the sensory input (e.g., motivation and reward), trigger caregiving, regulate emotions, and stimulate cognitions (e.g., attention, empathy, etc.). That is, infant cues are processed at different levels, and brain reactions regulate infant care.

## Limitations

The present experiments have several limitations. First, the small sample size is at the lower limit for between-subjects investigations of sex differences. Second, in the control experiment we used a different group of participants; for this reason we cannot draw certain conclusions regarding the specificity of female participants' responses to original baby cries and physically matched non-cry stimuli. Third, the sample was not homogeneous in terms of age (we considered participant age as a covariate in our analyses and found no significant effects of age). Fourth, we ruled out the possibility that some participants were professionally exposed to young children (kindergarten educators, teachers, professional baby-sitters), but we have no information on the other kinds of experience with children. Similarly, we have no information on other important variables that may influence responsiveness to baby cries, such as mood, empathy, or menstrual phase. A fifth limitation is the different influences on male versus female roles in society, where girls are generally prompted from a very early age to maternal roles and attitudes during play and social relationships. These cultural aspects do not allow us to draw strong conclusions about the biological versus cultural bases of the sex differences we observed. Finally, we had no behavioral characterization of the reflex response. Although we are inclined to interpret the MEPs to infant cry as indicating approach and protective behaviors toward an infant in distress, they could index any other (even aggressive) behavior. Further research is needed to clarify this issue.

## Conclusion

Caregiving behaviors in response to infant distress vocalization, especially motor responses, are observed in a wide variety of mammalian species. The present experiments extend this observation by providing evidence for the automaticity of motor cortex excitability in adult humans exposed to infant cries.

This response may be considered specific to baby cries because it was attenuated in baby cries with an increased fundamental frequency and absent to non-cry stimuli physically matched to natural infant cries (although more studies should clarify this aspect using within-group comparisons).

Finally, the finding of gender differences in automatic motor responses to infant cries suggests that females may to be tuned to respond to infant cries. Considering the importance of mothers' reactions to distressing stimuli produced by infants in predicting child outcomes (McElwain and Booth-LaForce, 2006; Leerkes et al., 2011; Joosen et al., 2012), this result may begin to explicate processes that regulate the quality of the mother-infant interactions and thereby possibly improve interventions aimed to promote positive and sensitive caregiving.

### Conflict of interest statement

The authors declare that the research was conducted in the absence of any commercial or financial relationships that could be construed as a potential conflict of interest.
